# Rediscovery of Older Adults’ Life Wisdom: Application of Narrative Therapy Using a Tree-of-Life Metaphor

**DOI:** 10.1093/geroni/igaa057.3059

**Published:** 2020-12-16

**Authors:** Chow Oi Esther

**Affiliations:** City University of Hong Kong, Kowloon, China

## Abstract

Using narrative therapy with a “Tree of Life” metaphor, practitioners facilitated 144 Hong Kong Chinese older adults through 24 4-session groups to rediscover their preferred identity and celebrate their lives. Thematic analysis of their ‘Tree of Life’ drawings revealed five themes: 1) Insights about their abilities, intentions and problem-solving capabilities; 2) A sense of purpose and commitment to their preferred identities; 3) Realization of personal values and beliefs; 4) Reconnecting sense of agency and hope; and 5) Cherishing life as a journey to celebrate their wealth of wisdom for common good. Despite confronting life challenges, participants re-author their sense of self consistent to their preferred identity, and renew purpose of life through drawing and dialectic narrative conversations. These findings depict a deeper understanding of older adults’ lives, and have significant theoretical and practical implications for health and social care professions to advance a constructive perspective to late life development.

